# Psychosis as a rare neuropsychiatric manifestation of Bardet–Biedl syndrome: A case report

**DOI:** 10.1177/03000605251379249

**Published:** 2025-09-24

**Authors:** Selebogo Moremi, Mpho Hubona, Taboka Maphorisa, Anthony A Olashore

**Affiliations:** Department of Psychiatry, 54547 University of Botswana, Botswana

**Keywords:** Bardet–Biedl syndrome, case report, rare disease, ciliopathy, hallucinations, delusions, psychosis

## Abstract

Bardet–Biedl syndrome is a rare, pleiotropic genetic disorder. Despite advances in genetic testing, the diagnosis of Bardet–Biedl syndrome remains primarily clinical, particularly in low-resource settings. Clinical features are classified as either primary or secondary. The primary features include retinal dystrophy, central obesity, renal abnormalities, male hypogonadism, and learning disabilities. Secondary characteristics include developmental delay, dental defects, diabetes mellitus, speech disorders, brachydactyly/syndactyly, and ocular abnormalities such as nystagmus, strabismus, and astigmatism. A significant proportion of patients with Bardet–Biedl syndrome present with behavioral and psychiatric symptoms. It is estimated that one-third of patients with Bardet–Biedl syndrome meet the criteria for a major psychiatric disorder during their lifetime. However, there is a paucity of research on neuropsychiatric traits and their management in Bardet–Biedl syndrome. Herein, we report a case of psychosis in a patient with features of Bardet–Biedl syndrome and describe the multidisciplinary management provided in a psychiatric setting.

## Introduction

Bardet–Biedl syndrome (BBS) is a rare autosomal recessive genetic ciliopathy affecting multiple organ systems.^1^ BBS exhibits genetic heterogeneity, with more than 26 genes identified to date; these genes localize to cilia, affecting their processes and multiple biological roles.^2^ Cilia are cellular structures present on most vertebrate organ cells. Pleiotropy in BBS results from widespread cilia expression and the diverse roles of cilia across different tissues.^3^ Defects in the primary (nonmotile) cilia of proliferating cells in photoreceptors, kidneys, limbs, and adipocytes lead to the anomalies observed in BBS.^4,5^ Other ciliopathies, such as Alström syndrome, exhibit clinical overlap with BBS in both phenotype and genotype; hence, they represent important differential diagnoses.^3^ As stated previously, BBS is a relatively rare condition, with a prevalence of approximately 1 in 160,000 within Northern European populations.^2^ The prevalence is higher in isolated communities (ranging from 1:13,500 to 1:17,500), such as those in Kuwait and Newfoundland, presumably due to prevailing consanguinity.^3^

Despite advances in molecular genetics, diagnosis remains primarily clinical, based on the clinical criteria developed by Forsythe and Beales.^2^ According to these criteria, a diagnosis is made when at least four of the following six primary features are present: retinal cone dystrophy, obesity, postaxial polydactyly, renal anomalies or dysfunction, cognitive impairment, and hypogonadism.^4^ Alternatively, diagnosis can be based on three primary and two minor features. Minor features include speech delay, diabetes mellitus, dental anomalies, ataxia, syndactyly, brachydactyly, and developmental delay.^4^ Ocular symptoms typically present in the first decade, while other features such as polydactyly or obesity may appear earlier.^3^ Retinal dystrophy (atypical retinitis pigmentosa) is the most characteristic feature, occurring in 94% of cases.^4^ Renal abnormalities significantly contribute to the mortality and morbidity associated with the syndrome. They include structural issues—such as cysts, dysplasia, and persistent fetal lobulation—and impaired renal function and may progress to end-stage renal failure.^3,6^ Hypogonadism may manifest as hypogenitalism/delayed puberty in males, and as urinary tract abnormalities in females.^3^ Postaxial polydactyly is a distinct feature of the syndrome that may be the only symptom present at birth.

Obesity is another significant characteristic of the syndrome.^3^ Although birth weight is usually normal, significant weight gain typically occurs within the first few months.^3^ Moreover, some BBS patients may develop diabetes mellitus type 2 in the second or third decade of their life, accompanied with other features of metabolic syndrome.^3^ Other organ systems, including the cardiovascular, gastrointestinal, endocrine (thyroid), and nervous systems, may also be affected.

Behavioral and psychiatric symptoms have been reported in BBS.^1^ These symptoms are concerning because they introduce complexity to the management of the disorder.^7^ Commonly reported symptoms, although often anecdotal, include cognitive impairment, obsessive–compulsive traits, autistic-like features, emotional immaturity, inability to recognize social cues, mannerisms (unusual gestures or vocalizations), frequent volatile anger outbursts, and shallow affect.^1,8,9^ Delirious mania, depression, and psychosis have also been reported, although the data remain limited.^5,7,10^ In a cohort of 109 BBS patients, Beales et al. observed that 2% had schizophrenia and an additional 5% had depression.^11^ In the same vein, Barnett et al.^1^ reported that 28% of the 21 children with BBS exhibited perceptual disturbances, including visual and auditory hallucinations.

Furthermore, data from animal model studies highlight the importance of ciliary pathways in the development and regulation of neuronal networks in the brain.^12,13^ The investigation and reporting of behavioral and psychiatric phenotypes in ciliopathies, particularly in the context of BBS, are essential. Documenting the behavioral phenotype adds to the currently limited literature and provides valuable insights for both caregivers and clinicians in the management of psychiatric symptoms associated with BBS. This study aimed to contribute to the understanding of psychiatric disorders in BBS; it describes the case of a patient who presented to our psychiatric facility with features of BBS and psychosis as well as the multidisciplinary management provided in this case.

## Case report

A female patient in her late 20s presented to the outpatient department of Sbrana Psychiatric Hospital, Lobatse, Botswana, at the end of 2022. She is the only child of nonrelated parents, with her father playing no role in her upbringing. According to her mother, the patient exhibited social withdrawal, persecutory delusions, and both visual and auditory hallucinations, despite her blindness. She frequently spoke to herself, reported seeing wild animals and hearing hostile voices, and displayed aggressive outbursts toward family members.

The patient had no history of psychoactive substance use, and there was no family history of psychiatric illnesses. A review of the medical history showed that psychiatric symptoms had not been observed before this presentation, excluding the learning difficulties experienced since childhood. Consequently, the patient was enrolled in special education from the age of 7 years until the onset of psychiatric symptoms. She was born at full term via cesarean section due to breech presentation to a 25-year-old mother, following an uncomplicated pregnancy. Her birth weight was recorded at 3150 g. She gained weight rapidly in infancy and was overweight by 2 months of age. At birth, she presented with postaxial polydactyly of the left hand and an incompletely formed postaxial appendage on the right hand; both digits were surgically removed during early childhood. Developmental delays in both speech and gross motor milestones were noted in early childhood.

At the age of 5 years, the patient was diagnosed with rod-cone dystrophy, also known as retinitis pigmentosa, by an ophthalmologist after presenting with diminished night vision. Her visual acuity gradually declined to complete blindness by her late teens. She has been unable to work or live independently, relying on her mother for daily assistance.

**Figure 1.**
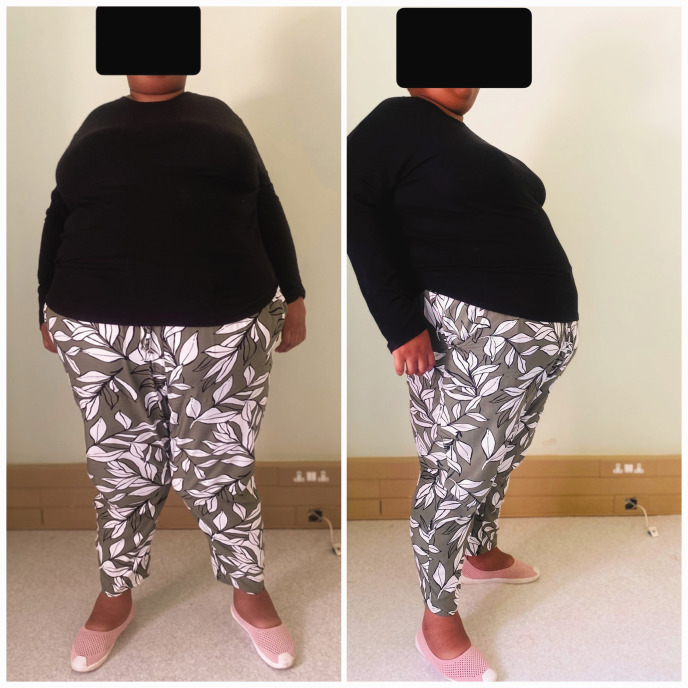
Central obesity.

**Figure 2.**
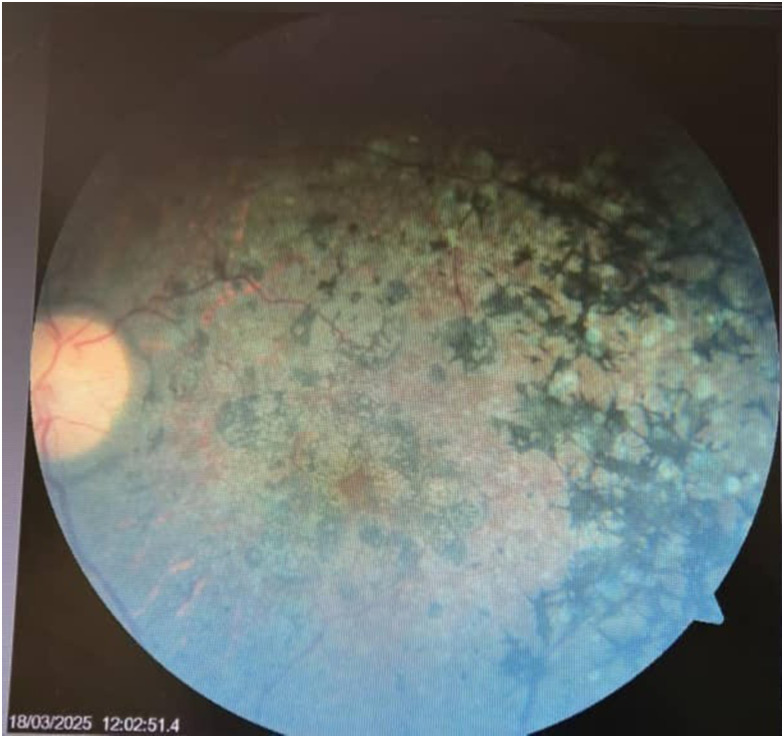
Retinal image depicting atypical retinitis pigmentosa.

On examination, she had obvious central obesity (Figure 1), with a weight of 135 kg and a height of 1.63 m. This corresponded to a body mass index (BMI) of 50 kg/m^2^, consistent with class III/extreme obesity according to the World Health Organization BMI classification. A surgical excision scar was noted on her left hand (for an extra digit). Micrognathia was noted in the absence of facial dysmorphic features. Horizontal nystagmus was observed in both eyes during ophthalmic examination. Retinal examination by the ophthalmologist revealed bull’s eye maculopathy, pale discs, attenuated vessels, and bony speckles, which are characteristic of atypical retinitis pigmentosa (Figure 2). Mental status examination was challenging owing to thought disorder (poverty of thought). Her vital signs, including blood pressure, heart rate, and respiratory rate, were within normal limits. Systemic examinations, including neurological, respiratory, and cardiovascular assessments, revealed no abnormalities.

Laboratory investigations, including a full blood count, renal function tests, electrolytes, and most liver function test parameters, were all within normal ranges, except for abnormalities in the lipid profile and gamma-glutamyl transferase (GGT) levels. The lipid profile revealed elevated cholesterol and triglycerides (Table 1). Her thyroid-stimulating hormone and thyroxine levels were normal, while triiodothyronine level was slightly low (Table 1). GGT was elevated (Table 1). Abdominal ultrasound showed the absence of the left kidney from the left renal fossa, with the right kidney measuring 13.5 × 6.6 cm. The ultrasound also demonstrated grade III fatty liver, with marked parenchymal hyperechogenicity and poor visualization of intrahepatic vessels and the diaphragm. There were no abnormalities on the pelvic ultrasound. No abnormalities were noted on the electrocardiogram. Molecular genetic tests were not performed, as they were not available in the country.

**Table 1.** Laboratory results.

Test name	Result	Reference range
Total cholesterol	6.2 mmol/L	3.6–5.7 mmol/L
Triglycerides	3.2 mmol/L	0.70–2.26 mmol/L
Triiodothyronine (T3)	1.01 nmol/L	1.3–3.1 nmol/L
Gamma-glutamyl transferase (GGT)	118 U/L	3–55 U/L

Psychosis was diagnosed according to the Diagnostic and Statistical Manual of Mental Disorders, Fifth Edition (DSM-5) criteria, in the context of an underlying diagnosis of BBS. The diagnosis of BBS was made clinically, as the patient exhibited five primary features and three secondary features, meeting the diagnostic criteria for BBS. Differential diagnoses also included other rare genetic disorders, such as Prader–Willi syndrome, Cohen syndrome, and Alström syndrome. Although these syndromes share features such as obesity and developmental issues with BBS, the presence of polydactyly and renal abnormalities in our patient led to the diagnosis of BBS.

She was initially started on olanzapine (5 mg daily) to treat her psychotic symptoms. She was later switched to quetiapine (600 mg daily) due to concerns about her metabolic profile; aripiprazole, which would have been an ideal option, was not available. Her psychotic symptoms improved within 3 months.

After approximately 1 year, her psychotic symptoms—characterized by paranoid delusions as well as visual and auditory hallucinations—recurred. She was treated with quetiapine at a maximum dosage of 800 mg; however, there was no remission of symptoms after an adequate duration. Consequently, she was transitioned to risperidone (2 mg daily), an alternative antipsychotic, which led to significant improvement in her condition after approximately 2 months. A multidisciplinary team—comprising a dietician, endocrinologist, ophthalmologist, and primary healthcare physician—was involved in the management of her condition. The dietician recommended dietary modifications and exercise routines, considering the patient’s obesity and abnormal lipid profile. Notably, the patient’s visual impairment limited her ability to monitor her diet, exercise, and medications independently. However, her mother, as the primary caregiver, played a crucial role in ensuring adherence to dietary instructions and prescribed medications.

The endocrinologist advised that given the normal thyroid-stimulating hormone levels, both thyroid hormones were likely normal; hence, the patient only required routine screening. Additionally, the surgical team assessed the patient and suggested bariatric surgery as a management option; however, she declined due to fear of complications. Subsequently, the patient developed diabetes mellitus and was prescribed metformin (1 g daily) as an oral antihyperglycemic medication. The onset of diabetes mellitus could be attributed to second-generation antipsychotics, a part of BBS, or unrelated factors. The antipsychotics prescribed are well-known to cause metabolic disorders, including diabetes.^14^ Furthermore, these medications are known to cause weight gain, which may have undermined the patient’s efforts to reduce weight.^14^ Other adverse effects, such as tremor and dyskinesias, were not reported during the treatment.

Education and counseling regarding psychosis, along with behavioral support therapy, were provided to both the patient and her mother. The patient continues to be monitored regularly by the local physician, dietitian, and psychiatric team, and she has remained free of psychotic symptoms for approximately 2 years. The patient and her mother expressed gratitude for the relief of her psychotic symptoms and the assistance they received from the facility. Furthermore, at the time of preparing this report, the patient had recently been admitted to a local rehabilitation center for the blind, which provides training in enhancing occupational and social functioning.

## Discussion

BBS is a rare, genetic, multisystem disorder, most commonly inherited in an autosomal recessive pattern.^2^ Its prevalence is higher in isolated communities.^3^ In our setting, the prevalence remains unknown; to the best of our knowledge, based on available literature and local clinical records, this is the first reported case of BBS in Botswana. The scarcity of data regarding BBS in Botswana may be due to underdiagnosis, stemming from a low index of suspicion or a lack of knowledge about the disorder. Another possible explanation could be the pleiotropic and variable onset of BBS, with the slow emergence of symptoms.^15^ This gradual and variable presentation makes it difficult for clinicians to detect such unique cases.

BBS is a clinical diagnosis characterized by genetic heterogeneity, with more than 26 BBS genes identified to date.^2^ Thus, BBS can be confirmed through genetic testing. If such tests are unavailable, as in our case, the diagnosis may be established based on the convincing clinical phenotype. Our patient presented with five of the six primary criteria: central obesity, polydactyly, rod/cone dystrophy (atypical retinitis pigmentosa), learning disability, and kidney abnormalities (unilateral renal agenesis). Moreover, our patient exhibited secondary features, including developmental delays, diabetes mellitus, and eye abnormalities (nystagmus). The patient was initially diagnosed with Prader–Willi syndrome. However, despite the presence of overlapping features, such as obesity and intellectual disability, the identification of polydactyly and retinal dystrophy primarily supported the diagnosis of BBS over Prader–Willi syndrome. This case highlights the importance of evaluating other syndromic disorders and considering the spectrum of ciliopathies in atypical clinical presentations.

Obesity is a common early feature of BBS, which was present in our patient.^16^ Obesity should be addressed through a multidisciplinary approach. Therapeutic agents, such as setmelanotide, have been shown to improve key BBS-related symptoms, including insatiable hunger and obesity, in a previous survey.^17^ Our patient was born with polydactyly, which was surgically excised in childhood. Retinal dystrophy was present in our case since childhood and progressed to blindness in late adolescence. Renal abnormalities occur in up to 90% of BBS cases.^3^ In our patient, unilateral agenesis was reported. Interestingly, at the time of preparing this report, renal function tests were within normal limits.

Behavioral, neurocognitive, and psychiatric symptoms have been reported in BBS, although the available data are limited.^8^ Such symptoms add to the complexity of management in BBS, particularly in a low-resource setting. Ciliary pathways have been implicated in the development and regulation of brain neural networks,^12,13^ which may explain the neuropsychiatric symptoms observed in ciliopathies such as BBS. In addition, our patient had intellectual disability and developmental delays. Of particular significance, in this case, the patient’s presenting complaint was psychotic symptoms, including delusions, hallucinations, and social withdrawal. This presentation is consistent with the observation that 2% of BBS cases exhibit psychotic symptoms,^11^ as described in a recent case report.^7^ The absence of a family history of mental disorders reduced the likelihood of primary psychotic disorders, which are often familial.

Psychiatric symptoms compound both functional impairment and the complexity of management in BBS.^7^ In our case, psychiatric symptoms imposed a significant burden on both the patient and her mother, as evidenced by social withdrawal and poor relations with other family members. For the clinical team, the patient’s intellectual disability made communication and further symptom elicitation challenging. The psychiatric symptoms in this case underscore the importance of considering underlying rare genetic disorders, particularly when they co-occur with obesity, intellectual disability, visual impairment, renal abnormalities, and polydactyly.

The metabolic profile of BBS (obesity, hyperlipidemia, and diabetes mellitus) made the selection of appropriate antipsychotics challenging. Aripiprazole, which has better metabolic-sparing effects, was not available at the time of the patient’s initial presentation. Nevertheless, treatment with alternative psychotropics and the involvement of other specialties (endocrinology, dietetics, and ophthalmology) was beneficial. However, her blindness made it difficult to adhere to regular exercises and diet modifications, as advised by the dietitian. This further underscores the difficulties in addressing comorbidities in BBS and the need for a multidisciplinary approach.

A key limitation in this case is the lack of genetic testing, although the clinical phenotype was convincing. Information on the specific mutation involved could contribute to existing data on genes responsible for BBS and their association with psychiatric symptoms. Therefore, genetic testing remains important and should be pursued when feasible, although it may not always be accessible in low-resource settings.

## Conclusion

Psychiatric symptoms contribute significantly to the complexity of the management of patients with BBS. This case report deepens our understanding of psychiatric symptoms in rare genetic disorders, particularly BBS, and explores their potential management. Clinicians should maintain a high index of suspicion when evaluating patients who present with psychiatric symptoms in conjunction with syndromic features. In particular, psychiatrists and other practitioners should always consider rare genetic disorders in patients who present with psychiatric symptoms accompanied with atypical physical features.

## Data Availability

The data supporting the findings of this report are available from the corresponding author on reasonable request.
